# Short Communication: Subtyping of *Mycobacterium kansasii* by PCR-Restriction Enzyme Analysis of the *hsp65* Gene

**DOI:** 10.1155/2013/178725

**Published:** 2013-12-22

**Authors:** Zofia Bakuła, Aleksandra Safianowska, Magdalena Nowacka-Mazurek, Jacek Bielecki, Tomasz Jagielski

**Affiliations:** ^1^Department of Applied Microbiology, Institute of Microbiology, Faculty of Biology, University of Warsaw, I. Miecznikowa 1, 02-096 Warsaw, Poland; ^2^Department of Internal Medicine, Pneumonology, and Allergology, Medical University of Warsaw, Żwirki i Wigury 61, 02-091 Warsaw, Poland; ^3^Clinic of Internal Medicine, Pneumonology, and Allergology, Independent Public Central Clinical Hospital, S. Banacha 1A, 02-097 Warsaw, Poland

## Abstract

*Mycobacterium kansasii* is one of the most common causes of pulmonary disease resulting from nontuberculous mycobacteria (NTM). It is also the most frequently isolated NTM species from clinical specimens in Poland. The aim of this study was to investigate the distribution of *M. kansasii* subtypes among patients suspected of having pulmonary NTM disease. Fifty clinical isolates of *M. kansasii* recovered from as many patients with suspected mycobacterial lung disease between 2000 and 2010 in Poland were genotyped by PCR-restriction enzyme analysis (PCR-REA) of partial *hsp65* gene. *Mycobacterium kansasii* subtype I was the only genotype to be identified among the isolates, both disease-associated and non-disease-associated. Isolation of *M. kansasii* subtype I from clinical specimens may be indicative of infection but may also merely represent colonization.

## 1. Introduction

*Mycobacterium kansasii*, a non tuberculous mycobacterium (NTM), is an opportunistic pathogen that causes both pulmonary and extrapulmonary infections [1–3]. As with other NTM, *M. kansasii* infections are believed to be acquired from environmental exposure rather than by human-to-human transmission. The natural reservoir of *M. kansasii* remains largely unknown. Rarely have the bacteria been isolated from soil, natural water systems, or animals. Instead it has quite often been recovered from municipal tap water, which is considered to be its major environmental source [4]. *Mycobacterium kansasii* is one of the most frequent NTM pathogens isolated from clinical samples throughout the world [1, 5–7]. According to a recent study on the global prevalence of NTM species, *M. kansasii* was the sixth most frequently isolated NTM. When focused on Europe, Poland, Slovakia, and the United Kingdom had the highest *M. kansasii* isolations of 35%, 36%, and 11%, respectively, compared to a mean isolation of 5% in Europe [1]. In most places, *M. kansasii* ranks second, behind only *Mycobacterium avium* complex, as a cause of NTM lung disease [8]. The annual rates of infection due to *M. kansasii* reported in the general population fall within the range of 0.2 to 0.3 cases per 100 000 [4], yet significant geographical variability exists [9–12]. In Poland, among the cases of NTM disease, whose number has been increasing remarkably in recent years, those attributable to *M. kansasii* are in the majority [13]. One in three NTM species isolated from patients with pulmonary mycobacterial infections is *M. kansasii* [1, 13].

Several molecular analyses have demonstrated that *M. kansasii* is a heterogeneous species [14–19]. To date, seven *M. kansasii* subtypes (I–VII) have been identified by PCR-restriction enzyme analysis (PCR-REA) of the *hsp65* gene [20]. The heterogeneity within the *M. kansasii* species has important clinical and epidemiological implications. There are reports that *M. kansasii* isolates that are involved in human disease belong almost exclusively to types I and II, with the former being the most commonly described [17, 20, 21].

The aim of this study was to determine the distribution of *M. kansasii* subtypes among 50 patients suspected of having pulmonary NTM disease.

## 2. Material and Methods

### 2.1. Strains

A total of 50 *M. kansasii* strains isolated from 50 patients with suspected *M. kansasii* infection (32 women and 18 men; median age: 64.6 ± 18.8 years; age range: 27–92 years), collected between 2000 and 2010 at the Department of Internal Medicine, Pneumonology, and Allergology of the Medical University of Warsaw, were included in the study. Patients were classified as having an infection in accordance with the criteria of the American Thoracic Society (ATS) [4]. The strains were cultured from sputa (28), bronchial washings (18), bronchoalveolar lavage fluids (3), and bronchial lavage fluid (1). The clinical samples were liquefied and decontaminated using soda lye with N-acetylcysteine and sodium citrate (final concentration: 2% NaOH, 0.5% NAC, and 1.3% C_6_H_5_O_7_Na_3_). The samples were then concentrated and cultured on Löwenstein-Jensen (L-J) medium. The isolates were identified as *M. kansasii* by using the high pressure liquid chromatography (HPLC) methodology, in accordance with the Centers for Disease Control and Prevention (CDC) guidelines [22].

### 2.2. DNA Extraction

Genomic DNA was extracted using Amplicor Respiratory Specimen Preparation Kit (Roche Diagnostics, Switzerland) as described elsewhere [23].

### 2.3. Amplification and Restriction Analysis

For the amplification of a 441 bp fragment of the *hsp65* gene Tb11 and Tb12 primers were used, as described by Telenti et al. [15]. The PCR mixtures were prepared with a TopTaq Master Mix kit (Qiagen) in a final volume of 50 *μ*L containing ca. 10 ng of genomic DNA. After initial denaturation at 94°C for 3 min, the reaction mixture was run through 35 cycles of denaturation at 94°C for 30 s, annealing at 57°C for 30 s, and extension at 72°C for 30 s, followed by a final extension at 72°C for 10 min. Amplified fragments were digested with HaeIII and Eco91I (BstEII) restriction enzymes (FastDigest), under conditions recommended by the manufacturer (ThermoScientific), separated by electrophoresis in 4% agarose gels, and visualized by staining with ethidium bromide (0.5 *μ*g/mL) and exposure to UV light (*λ* = 320 nm).

Strains were classified into subtypes based on their PCR-REA patterns obtained in two separate PCR-REA assays.

## 3. Results and Discussion

Of the 50 patients under the study, 23 (46%; 15 women, 8 men aged 56.9 ± 20.3 years; age range: 27–87) met the ATS criteria for the definition of *M. kansasii* disease. For the remaining 27 (53%) patients, the NTM case definition criteria, either clinical or bacteriological, were not fulfilled.

**Figure 1:**
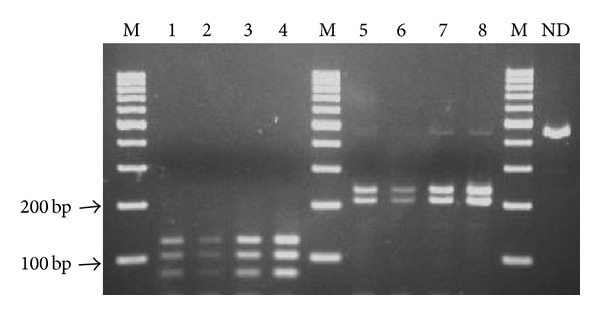
Differentiation of *M. kansasii* subtypes by PCR-REA of *hsp65*. Amplified *hsp65 *fragments were digested with HaeIII (lanes 1–4) and BstEII (lanes 5–8). Lanes: M: GeneRuler 100 bp DNA Ladder (ThermoScienific), ND: nondigested fragment of *hsp65*.

All the *M. kansasii* isolates tested yielded, upon PCR amplification of partial *hsp65* gene, a single product of expected size (ca. 440 bp). When subjected to restriction endonuclease digestion with the enzyme HaeIII, the amplicons always produced three DNA fragments of 140, 105, and 80 bp in length. Likewise, digestion of the amplified *hsp65* fragment with BstEII yielded each time the same two-band pattern (fragments of 240 and 210 bp in length) (Figure 1). According to PCR-REA patterns obtained in two different PCR-REA assays, all the 50 *M. kansasii* isolates were categorized into type I.

Poland is the country with the highest *M. kansasii* isolation rate in Europe (35% of all NTM isolations in Poland compared to a mean isolation rate of 5% for Europe) [1]. This study is the first to document the distribution of *M. kansasii* genotypes among patients with pulmonary disease from Poland.

The reported results are consistent with those of previous studies. The investigations performed so far have suggested that *M. kansasii* type I is the most prevalent type from clinical isolates worldwide. The distribution of the genotypes (subtypes) among *M. kansasii* isolates was first studied by Picardeau et al. in the late 1990s [17]. Of the five (I–V) recognized genotypes, genotype I was the most common and included 25 (39.7%) of the 63 *M. kansasii* isolates, of both environmental and clinical origin. Among the latter group, all five genotypes were encountered with only 16 (42.1%) of the 38 isolates being differentiated into genotype I. The frequency of this genotype was found to be much higher in a study of Alcaide et al. [24]. *Mycobacterium kansasii* subtype I was present in 109 (66.9%) of the 163 clinical isolates from different European settings. A higher percentage of type I *M. kansasii* clinical isolates was found in three subsequent European studies. Taillard et al. reported 77.9% (60/77) of the isolates from Switzerland belonging to genotype I [20], whereas in a study by Gaafar et al., of the 252 *M. kansasii* isolates collected in Spain, only two belonged to genotype II, with all the remaining isolates being representatives of genotype I [19]. Another Spanish study revealed the absence of genotype II among *M. kansasii* clinical isolates, with 91 (97.8%) of the 93 isolates tested representing genotype I and the remaining two isolates representing genotype VI [25]. An analysis of human *M. kansasii* isolates from the United States showed that all but three (78 of 81 isolates) belonged to subtype I. Of the remaining three isolates, two belonged to subtype III and one belonged to subtype II [26]. Similar results were obtained by Chimara et al. in Brazil, where out of 184 patient isolates of *M. kansasii* only two were other than type I isolates (one belonged to type II and the other to type III) [27].

Some authors suggest that in the absence of complete clinical information on patients from whom *M. kansasii* isolates are obtained the PCR-REA analysis of the *hsp65* gene may be useful in categorizing isolates as associated with mycobacterial disease (types I and II). However, as evidenced in our study, recovery of *M. kansasii* type I isolates from clinical samples does not necessarily correlate with clinical picture. This has also been observed by others [20, 28]. Isolation of *M. kansasii* subtype I from clinical samples may be indicative of infection but may also merely represent colonization.

## 4. Conclusions

To conclude, *M. kansasii* subtype I was the only subtype recognized among the 50 *M. kansasii* isolates, both disease-associated and non-disease-associated. High detection rate of *M. kansasii* subtype I in clinical samples may suggest that this genotype has a particular propensity for colonization, and thus a higher epidemiological potential for humans. More comprehensive studies, on large collections of *M. kansasii *isolates, are needed to provide a better understanding of the biology and pathogenicity of *M. kansasii* subtype I. An important consideration to be addressed in these studies is the possible high degree of heterogeneity of *M. kansasii* type I isolates.
